# Performance of on-site Medical waste disinfection equipment in hospitals of Tabriz, Iran

**DOI:** 10.15171/hpp.2016.33

**Published:** 2016-10-01

**Authors:** Hassan Taghipour, Mina Alizadeh, Reza Dehghanzadeh, Mohammad Reza Farshchian, Mohammad Ganbari, Mohammad Shakerkhatibi

**Affiliations:** ^1^Department of Environmental Health Engineering, Tabriz University of Medical Sciences, Tabriz, Iran; ^2^East Azerbaijan Health Center, Tabriz University of Medical Sciences, Tabriz, Iran

**Keywords:** Medical waste, Hospitals, Disinfection, On-site, Equipment, Evaluation

## Abstract

**Background:** The number of studies available on the performance of on-site medical waste treatment facilities is rare, to date. The aim of this study was to evaluate the performance of onsite medical waste treatment equipment in hospitals of Tabriz, Iran.

**Methods:** A various range of the on-site medical waste disinfection equipment (autoclave, chemical disinfection, hydroclave, and dry thermal treatment) was considered to select 10 out of 22 hospitals in Tabriz to be included in the survey. The apparatus were monitored mechanically, chemically, and biologically for a six months period in all of the selected hospitals.

**Results:** The results of the chemical monitoring (Bowie-Dick tests) indicated that 38.9% of the inspected autoclaves had operational problems in pre-vacuum, air leaks, inadequate steam penetration into the waste, and/or vacuum pump. The biological indicators revealed that about 55.55% of the samples were positive. The most of applied devices were not suitable for treating anatomical, pharmaceutical, cytotoxic, and chemical waste.

**Conclusion: ** Although on-site medical waste treating facilities have been installed in all the hospitals, the most of infectious-hazardous medical waste generated in the hospitals were deposited into a municipal solid waste landfill, without enough disinfection. The responsible authorities should stringently inspect and evaluate the operation of on-site medical waste treating equipment. An advanced off-site central facility with multi-treatment and disinfection equipment and enough capacity is recommended as an alternative.

## Introduction

Medical waste include all the waste generated by hospitals, health care services, research facilities, and laboratories as major sources as well as the waste originated from minor health care services.^1^ The quantity of medical and health-care waste have, rapidly, been increased in recent decades due to the enhanced quality services, population growth, the number and size of health care facilities, as well as using disposable medical products.^2,3^ The generation rate of medical waste in Iran have been reported to be 2.71-4.45 kg/bed-day, and in the other countries, this amount is in the range of 0.84 to 7 kg/bed-day.^1,4,5^

Medical waste may be classified in two major groups: the general waste and the hazardous-infectious waste. About 75% of the waste produced by hospitals and 90% of those produced by health care services are general health care waste and the remaining 10%-25% is regarded as hazardous-infectious waste.^1,6^ Thus, the most of the general waste produced by hospitals and health care services, if properly segregated, do not need any special treatment and may be disposed through the current municipal waste management system.^7^

The hazardous-infectious medical waste include a variety of infectious, pathological, genotoxic, pharmaceutical, and chemical waste involving a high level of heavy metals, pressurized containers, and radioactive waste.^1,8^ Improper treatment and disposal of hazardous-infectious medical waste can cause serious risks to public health and environment.^1,9-11^

The treatment of the hazardous-infectious medical waste may be carried out on-site or off-site of the health care facilities.^1,6^ Each of the on-site or off-site medical waste treatment methods has its own advantages and disadvantages.

According to the Act 64 in the Iranian Medical Waste Management Regulations (IMWMRs), all the waste producers in the middle-sized and the large cities are responsible for treating their hazardous-infectious waste and converting them into the general waste in the on-site facilities.^12^ So, the most of hospitals (and the other major producers of the medical waste) in the country have selected the on-site treating method as the preferred one.^6^

Numerous studies have been carried out on the characterization, regulation, management, treatment, and disposal of the medical waste.^2,19,13-26^ However, the number of studies on the performance evaluation of the on-site medical waste treatment facilities in, both, developed and developing countries are rare to date. Therefore, the aim of this study was to evaluate the performance of on-site medical waste treatment equipment in the hospitals of Tabriz, Iran.

## Materials and Methods

### Study area and selection of the studied hospitals

Tabriz is the largest and the capital city of East Azerbaijan province in the northwest of Iran with the population of 1 545 491 people (2011) and the area of 237.45 km^2^. There are 22 hospitals in Tabriz with more than 3600 in-use beds. The hospitals in the city may be divided into the governmental, educational (university), private, nongovernmental organizations (NGOs as charity institutions), and military centers. Among the 22 hospitals, 10 were selected to be included in the evaluation process (Table 1). To do so, all the hospitals with on-site medical waste treatment equipment were ranked according to the number of in-use beds from the highest to the least and, then, a range was selected to represent a variety of the hospitals in terms of the number of in-use beds. As there is shown in Table 1, the evaluation was designed to cover the various sizes and categories of the hospitals with different kinds of medical waste treating equipment including autoclave, chemical disinfection, hydroclave, and dry thermal treatment. It should be noted that, based on the Act 70 of the IMWMRs, the application of any kind of incinerator is forbidden in the cities.^12^ Therefore, all the incinerators had been shut down.

### Evaluation of the on-site medical waste treatment equipment in hospitals 

The heads of the hospitals were invited to collaborate in the study and support the research team. The sites of the hospitals were visited to gather the basic information and to evaluate the working conditions of the facilities. In each hospital, the environmental health officer of the hospital was involved in the evaluation. At the beginning of the evaluation, all the research team involved in the study attended a training course to find a better understanding on the purpose and the correct procedures of the study. The data were collected from the hospitals applying a checklist and site visits (observational method). In 2014, during a six months period, the related equipment in all of the selected hospitals were mechanically, chemically, and biologically monitored. The mechanical monitoring included the control and recoding the physical criteria of the treating process of the equipment (temperature, pressure, detention time, amount of loading, etc.). The chemical and biological monitoring were conducted once per month without making any change in the regular operation of the facilities in all of the selected hospitals, except for one which was due to technical problems.

### Mechanical, chemical and biological monitoring methods

The mechanical inspection (temperature, pressure, detention time, amount of loading, etc.) as well as the chemical and biological monitoring were carried out according to the Iranian national guideline for evaluating the medical waste treatment equipment.^27^ During the study, 18 chemical and 54 biological monitoring tests were carried out. The chemical monitoring was conducted applying the Bowie-Dick test card only for the autoclaves. Bowie-Dick tests have been designed as an environmentally safe, lead-free method for daily monitoring of pre-vacuum steam sterilizers to detect air leaks, inadequate steam penetration, and vacuum pump failures.

Biological monitoring was performed through placing a vial biological indicator (VBI) and a strip biological indicator (SBI) inside the representative materials which were, previously, disinfected. VBI contained *Geobacillus stearothermophilus* spores (SAL≤ 10^6^ CFU/vail) and SBI contained *Bacillus atrophaeus* spores (SAL≤ 10^6^ CFU/srip). Both of these spores are among the most resistant kinds of spores.

VBI included a plastic vial with an inoculated spore strip and a sealed glass vial containing media placed inside. The outer vial was sealed with a plastic cap and a filter paper. The small orifices in each cap provided the steam penetration. After normal sterilizing process, when the glass vial was crushed, the growth media flowed into the inoculated spore strip. The vial was, then, incubated (at 56°C for 24 to 72 hours) and, if viable spores were present, the color of the media was altered.

In the case of SBI, after exposure, the glassine envelopes were aseptically opened and transferred to an individual tube containing 10 mL sterile casein soybean digest broth applying sterile forceps. The tubes were incubated for 24 to 72 hours days at 36°C. During the study, the tubes were, daily, observed for growth (medium turbidity = growth = non-sterile and clear medium turbidity = no growth = sterile). The control was carried out by including one or more positive controls in each series of the tests (as positive control) and also incubation of at least one unused tube of culture medium from the same batch/lot (as negative control). The results of the medical waste treating equipment that were not working during the study due to the lack of operation and maintenance were considered as positive, without placing biological indicators inside them. Data was presented using frequency (percentage) for categorical variables. All analyses were performed using SPSS 16 software (SPSS Inc., IL, Chicago, USA).

## Results

A summary of the current condition of the on-site medical waste treatment systems in the studied hospitals are presented in Table 2. Among all the selected hospitals, three were equipped with autoclaves, four with chemical disinfections, two with hydroclaves, and one with dry thermal treatment (Table 1). Moreover, all of the operators working with the medical waste treatment devices had passed special training courses. Only in four out of all the studied hospitals, the safety criteria had been considered for operating with the devices. Also, in five hospitals, suitable spaces had been allocated to the devices and the operators were satisfied with operating the installed equipment.

The mechanical monitoring of the treatment systems in the most of the hospitals indicated that the mechanical criteria (temperature, pressure, detention time, amount of loading, etc.) were according to the standard equipment operation procedures. The results of Bowie-Dick tests indicated that 38.9% of the installed autoclaves had operational problems in pre-vacuum, air leaks, inadequate steam penetration, or vacuum pump. In addition, about 28% of the medical waste treatment and disinfection equipment were not working due to some operational and maintenance problems, and thus, all the hazardous-infectious waste were sent out to the general waste treatment without any specific treatment. The results of the biological monitoring of the equipment in the studied hospitals are presented in Table 3. The rustles of biological indicators (VBI and SBI) indicated that about 27.21% of the medical waste samples treated by the installed equipment were positive. Considering that one of the equipment was not working (due to technical and operational problems), about 55.55% of the samples were positive.

**Figure 1:**
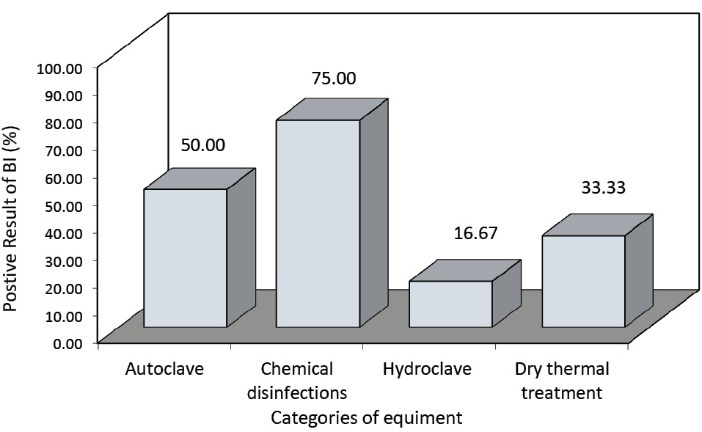
Comparison of the operation of the medical waste treatment equipment based on the positive biological test results.

**Figure 2:**
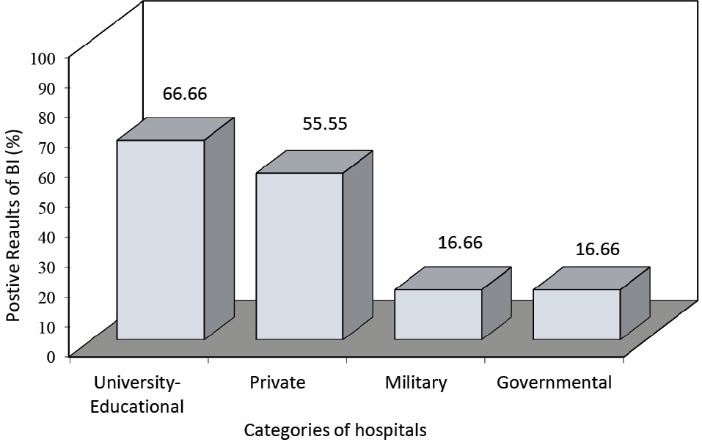
Comparison of the hospitals based on the positive biological test results

The comparison of the operation of the medical waste treatment equipment based on the biological test results is presented in Figure 1. Also the comparison of the hospitals based on the positive biological test results is presented in Figure 2.

**Table 1:** Summary of the characteristics of Tabriz hospitals sampled in the present study

**Hospital**	**Dependency**	**Activity**	**In-use beds, n**	**Treatment technology**
A		Specialized and subspecialized - General	517	Wet thermal (Autoclave)
B		Specialized and subspecialized - General	243	Wet thermal (Autoclave)
C	University-governmental	Specialized and subspecialized	186	Wet thermal (Hydroclave)
D	Educational-treatment	Specialized and subspecialized of woman (gynecology & Obstetrics) & IVF	127	Chemical disinfection
E		Specialized and subspecialized of Ophthalmology	66	Chemical disinfection
F	Social security-governmental	Specialized and subspecialized of woman	70	Dry thermal treatment
G	Private	General	250	Chemical disinfection
H	Private	General	75	Wet thermal (Autoclave)
I	Private	General-Specialized	50	Chemical disinfection
J	Military	General	100	Wet thermal (Hydroclave)

**Table 2:** Summary of the current condition of the on-site medical waste treatment systems in the studied hospitals

**No**	**Items**	** Answer choices**	
1	Appropriate usage of color-coded containers to segregate the medical waste	Good (80%)	Poor (20%)
2	The producer country of the treatment equipment	Iran (100%)	Foreign countries(0%)
3	Being equipped with parallel treatment system for emergency conditions (phasing out of the system due to technical problems)	Yes (10 %)	No (90%)
4	The number of required skilled operators	At least 2 operators (100%)	
5	Training the medical waste equipment operators (self-statement)	Yes (100%)	No (0%)
6	Reliability of the treating equipment according to the report of the operators	Yes (55.5%)	No (45.5%)
7	Accepting the treatment of the medical waste produced by the minor medical care systems in the city	Yes (0%)	No (100%)
8	Accepting the treatment of the anatomical, pharmaceutical, cytotoxic and chemical waste	Yes (0%)	No (100%)
9	The management quality of the area around the treating equipment	Good (55.5%)	Poor (45.5%)

**Table 3:** The result of biological monitoring of on-site medical waste treatment equipment

**Result**	**Months if monitoring**						**Average**
	**1**	**2**	**3**	**4**	**5**	**6**	
Positive^a^ (%)	77.77	55.55	44.44	44.44	55.55	55.55	55.55
Negative (%)	22.23	44.45	55.56	55.56	44.45	44.45	44.45

^a^The result of equipment that was not working during the study considered positive, without placing Biological Indicators inside of them.

## Discussion

About 80% of the studied hospitals had a proper program for segregating the infectious- hazardous waste from the general. In a previous study in 2009, Taghipour and Mosaferi reported the number of Tabriz hospitals equipped with a successful segregation program as 40%.^15^ A simple comparison of these two studies shows a remarkable improvement in the situation. In all of the on-site treatment facilities, one fulltime worker was, only, employed for operating with the equipment, while at least two fulltime highly skilled technicians were needed as operators of a device. Based on our results, the most of the installed devices were not suitable for treating anatomical, pharmaceutical, cytotoxic, and chemical waste. Therefore, such generated waste were being sent to the municipal waste collection and the general disposal stream. In addition, none of the studied hospitals accepted the treatment of the hazardous-infectious waste from the minor medical waste producers in the city. Inevitably, almost all of the minor medical waste producers had to send their hazardous-infectious waste to the municipal waste stream without any treatment for final disposal.

The most of hospitals in the present study had problems in financing, planning, operating, and maintaining the equipment, which was in agreement with the prior study in the region.^6^ In 90% of the hospitals, the treatment capacity was enough only for their own generated waste. Nevertheless, in 10% of the hospitals, there was not enough treatment capacity to cover their own generated waste. Furthermore, the most of hospitals (90%) were equipped only with one treatment equipment, so if the systems initiated any technical problem, all the hazardous-infectious waste would be sent out to the general treatment system without any special treatment.

According to the biological monitoring of the equipment in the studied hospitals, it was found that only about 44% of the on-site medical waste treating disinfection devices were in perfect use. While according to IMWMRs, the treating systems should have microbial inactivation reduction by 10^6^ colony forming unite.^12^ These findings indicate that about more than half of the devices in the studied hospitals does not have the least level of standards defined for the treatment systems. Because the highly infectious waste, such as cultures, stocks of infectious agents generated from laboratory works, and anatomical parts without any disinfection were being sent to the municipal landfill site, for final disposal. Such medical waste disposal may bring about lots of environmental and health concerns, considering that the general landfill sites were easily accessible to unauthorized people, which may result in great health risks for health-care stuff, municipal workers, the people, and the environment, as well. Moreover, the disposed medical waste in landfills may be, illegally, segregated and recycled along with municipal solid waste.^15^

The comparison of the operation of the medical waste treatment equipment based on the biological test results indicated that the chemical disinfection had the worst result as 75% of the samples were positive. The hydroclave showed the best result in which 16.67% of the samples were positive. As indicated in Figure 2, the biological test results of the equipment in the educational hospitals (university hospitals) private hospitals and military and governmental hospitals were 66.66%, 55.55%, and 16.66%, respectively.

Monitoring the on-site medical waste treatment systems in the 10 studied hospitals during six months revealed that despite the training courses provided for the equipment operators, the most of equipment had operation and maintenance problems. These findings urge the need for more stringent and stricter polices, rules, and regulations to be applied to all levels of the medical waste management in the hospitals. Efficient and proper training courses should be implemented for the hospital staff at different levels, especially those who work with the medical waste treating equipment. The ministry of health and, also, the Environment Protection Agency (EPA) should, stringently, supervise the on-site waste treating systems and the process of selecting and insulating the suitable and efficient equipment.

## Conclusion

Biological monitoring of the installed medical waste equipment in the hospitals indicated unacceptable condition in the present study. Also, the most of applied treatment devices were not suitable for treating anatomical, pharmaceutical, cytotoxic, and chemical waste. The most of on-site medical waste treating systems had operational and maintenance problems, indicating that the current condition of the on-site waste treating facilities results in wasting the capital and human resources. Therefore, the hospital managers should take more responsibility for more proper treatment of medical waste in the on-site facilities and should allocate enough funds and highly skilled technicians to enhance the operation of the equipment. Considering the current problems found in the on-site medical waste disinfection and treatment equipment of Tabriz hospitals, an advanced central off-site facility with multiple devices and enough capacity for treatment and disinfection processes is proposed as a suitable alternative. To do so, a pilot study on the central off-site system may be helpful.

## Acknowledgments

This study was funded by the Research Deputy of Tabriz University of Medical Science. The authors thank all of the members of the survey team and the hospitals that participated in the study.

## Ethical approval

Procedures were approved by the Research Vice-Chancellor of Tabriz University of Medical Sciences review board.

## Competing interests

None of the authors have any conflict of interest.

## Authors’ contributions

LD was involved in the conception of the study, performed data collection and the analyses and drafted the manuscript. RD was involved in the conception of the study, interpreted the results from the analyses, performed significant revisions, assisted in the revision of the manuscript and approved the final version of the manuscript.
